# Characterization of polyhydroxyalkanoate production capacity, composition and weight synthesized by *Burkholderia cepacia* JC-1 from various carbon sources

**DOI:** 10.1016/j.heliyon.2022.e09174

**Published:** 2022-03-24

**Authors:** Julian Hock-Chye Chin, Mohd Razip Samian, Yahaya M. Normi

**Affiliations:** a School of Biological Sciences, Universiti Sains Malaysia, 11800, Minden, Penang, Malaysia; b Enzyme and Microbial Technology (EMTech) Research Center, Faculty of Biotechnology and Biomolecular Sciences, Universiti Putra Malaysia, 43400, Serdang, Selangor, Malaysia; c Department of Cell and Molecular Biology, Faculty of Biotechnology and Bimolecular Sciences, Universiti Putra Malaysia, 43400, Serdang, Selangor, Malaysia

**Keywords:** *Burkholderia cepacia* JC-1, Glucose, Palm oil, Chicken fat, P(3HB), 3HV, Molecular weight

## Abstract

Polyhydroxyalkanoates (PHA) are microbial polymers that have received widespread attention in recent decades as potential alternatives to some petrochemical-based plastics. However, widespread use of PHA is often impeded by its cost of production. Therefore, the search for and systematic investigation of versatile microbial PHA producers capable of using various carbon sources, even in the form of animal fats, for PHA biosynthesis is desirable. This study highlights the PHA production capacity, monomer composition and molecular weight synthesized by *Burkholderia cepacia* JC-1, a locally isolated strain from soil, from various carbon sources. In the category of simple sugars and plant oils, the use of glucose and palm oil at C:N ratio of 40 resulted in the highest accumulation of 52 wt% and 36 wt% poly(3-hydroxybutyrate) [P(3HB)] homopolymer and dry cell weight of 2.56 g/L and 3.17 g/L, respectively. Interestingly, *B. cepacia* JC-1 was able to directly utilize animal-derived lipid in the form of crude and extracted chicken fat, resulting in appreciable dry cell weight and PHA contents of up to 3.19 g/L and 47 wt% respectively, surpassing even that of palm oil in the group of triglycerides as substrates. The presence of antibiotics (streptomycin) in cultivation medium did not significantly affect cell growth and polymer production. The supply of sodium pentanoate as a co-substrate resulted in the incorporation of 3-hydroxyvalerate (3HV) monomer at fractions up to 37 mol%. The molecular weight of polymers produced from glucose, palm oil and chicken fat were in the range of 991–2118 kDa, higher than some reported studies involving native strains. The results from this study form an important basis for possible improvements in using *B. cepacia* JC-1 and crude chicken fats in solid form for PHA production in the future.

## 1 Introduction

It has become common knowledge that the availability of biodegradable and environmentally friendly materials holds possible answers to the nagging issue of a sustainable environment. A low percentage recovery and pollution by recalcitrant solid materials like plastics is as palpable a problem as the depletion of petroleum reserves, the source from which plastics are derived from. The conventional, petrochemically derived plastics that the populace has grown dependent upon are useful and ubiquitous. The durability of the plastics allows them to persist in the environment for a long time. As plastics degrade slowly, this poses challenges to solid waste management [1]. Furthermore, its generation and consumption exceed recycling and further led to the accumulation of plastic wastes. To add to these problems, harmful leachates attributed to plastic wastes are detected in landfills [2] whilst the combustion of plastics results in the release of a host of health-hazardous compounds [3]. In addition, reduce, reuse and recycle practices may not always be possible and in some cases, impractical [4]. Based on United Nations Environment Programme (UNEP) statistics, only 9 per cent of plastics are recycled [5].

Biodegradable polymers possess physical properties similar to that of plastics and may complement, if not supersede, their daily usage. However, it is the inherence of biodegradability in these materials and the ability to synthesize them from various renewable carbon sources that made them such an interesting entity for research. PHA is one such material and is biologically synthesized by a wide range of bacteria during an imbalanced growth condition with an excess in carbon source [6]. Among the many classes of industrially relevant biopolymers synthesized by bacteria, only PHA and a few other polymers which are employed largely as food additives are in commercial production [7, 8, 9]. This is largely due to the beneficial properties of PHA, ranging from the biodegradability and biocompatibility to thermoplastic properties of the biopolymer [10, 11]. In general, PHA are classified into two groups based on the number of carbon atoms of the monomer unit. Short-chain-length PHA (SCL PHA) are comprised of 3–5 carbon atoms while medium-chain-length PHA (MCL PHA) are comprised of 6–14 carbon atoms.

Although PHA has been crowned as one of the promising alternatives to petroleum-based plastics, the main factor which impedes the production and large scale application of the polymer is the production cost caused mainly by the carbon feedstock used [7, 12]. Acknowledging the prospect of PHA polymer and the issues surrounding it, a suitable host capable of using renewable carbon sources, such as animal fat for PHA biosynthesis is desirable and systematic investigation to establish the extent of polymer biosynthesis by the host from various carbon sources is needed. Although production of PHA from various wastes such as glycerol, a by-product from biodesel industry [13, 14, 15] and food wastes [16] have been given attention of late, the use of crude animal fats as the main feedstock in particular, is still scarce [17, 18, 19, 20, 21, 22]. The scarcity on studies related to PHA biosynthesis from animal fats most probably due to difficulty or inability of microbial cells in breaking down solid fats during the cultivation process [23]. Therefore, the obtainment of microbial strains that are capable to directly utilize crude animal fats for PHA production is very much needed as this would eliminate the need to treat such feedstocks prior to PHA production and hence, would minimize the cost of PHA production.

This study highlights the PHA biosynthesis capabilities, composition and molecular weight of polymers produced by a locally isolated bacterial strain, *Burkholderia cepacia* JC-1 from various renewable carbon sources ranging from simple carbohydrates, plant oils and even crude chicken fats. Findings in this study showed that the strain was able to directly metabolize crude chicken fats for PHA production, yielding higher PHA content than that of palm oil. This study serves as a basis for possible future improvements in cultivation conditions and utilization of the strain in scaled up PHA production using crude chicken fats as the main, economical feedstock.

## 2 Materials and methods

### 2.1 Bacterial strain

The bacterium that was used throughout this study was a strain previously isolated from soil at Aman Lake, Universiti Sains Malaysia (5.3545° N, 100.2983° E) and identified as *Burkholderia cepacia* via 16S rDNA analyses (Accession No.: JQ080270) and biochemical profiling [24]. To distinguish the isolate from other types of microorganism of the same species-kind, the isolate is confirmed as *B. cepacia* JC-1.

### 2.2 PHA biosynthesis by *B. cepacia* JC-1

#### 2.2.1 Lipid extraction from adipose tissues of chicken

Adipose tissues were kept frozen at -20 °C until ready to be processed. Lipid extraction was carried out based on the Folch method [25]; as described by Shahidi (2003) [26]. Small cuts (50 g) of adipose tissue were weighed and homogenized in 100 mL methanol. Chloroform (50 mL) was then added and the mixture was homogenized for 2 min. An additional 50 mL of chloroform was added and homogenized for 30 s. Finally, 50 mL of water was added and homogenized for 30 s. The resulting homogenate was then centrifuged at 3000 × g for 5 min and the supernatant was decanted into a separatory funnel. The chloroform layer was passed through a 2.5 cm thick layer of anhydrous sodium sulphate using a Whatman Grade No. 1 filter paper in a funnel. A 20 mL mixture of 1:1 (v/v) chloroform/methanol was used to wash the layer of salt in the filter paper. The solvent was then removed in a rotary evaporator under vacuum at 40 °C. The weight of the extracted lipid and lipid content were then determined.

#### 2.2.2 Carbon sources

The substrates used as the carbon source for the biosynthesis of PHA can be divided into three main classes, viz. simple carbohydrates, triglycerides and sodium salt of organic (alkanoic) acids. Table 1 lists the substrates used as carbon source during cultivation as well as the amount incorporated into the medium. Both glucose and palm oil were used to represent their respective classes in determining the amount of carbon substrate to use as well as the cultivation time that will be optimum for growth and PHA biosynthesis. These parameters were then adopted for use by other members of the class. Stock solutions of both the simple carbohydrates and sodium alkanoates were prepared in distilled water and filter-sterilized through a 0.2 μm pore size membrane. The triglycerides were sterilized by autoclaving. All respective carbon sources were then aseptically added into the cultivation medium to the desired amount (Table 1). Frozen chicken adipose tissue was cut into single chunks and weighed. Chunks weighing about 2 g were used for the cultivation. The chunks of fat were handled with a flamed forceps, dipped into methanol, and then removed to allow for the evaporation of methanol. They were then added into the flasks similarly to other substrates used as the carbon source.

**Table 1:** Substrates used as carbon sources and their corresponding amount.

Carbon sources	Amount used
*Simple carbohydrates*	
Glucose	20 g/L^a^, C:N^b^ = 10–50
Fructose	The best C:N ratio based on the results of the cultivation on glucose
Galactose	
Sucrose	
Xylose	
*Triglycerides*	
Palm oil	2% (v/v)
Coconut oil	2% (v/v)
Soybean oil	2% (v/v)
Chicken fat solids	2 g
Extracted chicken fat	2% (v/v)
*Organic acids*	
Sodium hexanoate	1 g/L^a^, 1–6 g/L
Sodium octanoate	1 g/L^a^, 1–6 g/L
Sodium pentanoate	1–9 g/L^c^

a Initial amount used in the experiment to obtain the time profile for growth and PHA biosynthesis of *B. cepacia* JC-1.

b Molar ratio of carbon to nitrogen.

c Used as a co-substrate in conjunction with glucose (at a fixed C:N).

#### 2.2.3 Cultivation conditions

The cultivation in glucose and palm oil to obtain the time profile for cell growth and PHA biosynthesis was carried out in 400 mL of mineral salt (MS) medium (KH_2_PO_4_ (4.17 g/L), Na_2_HPO_4_ (2.75 g/L), NH_4_Cl (0.50 g/L), MgSO_4_ (100 mM), trace elements (0.1% v/v)). All other cultivations were carried out in Erlenmeyer flasks containing 100 mL of MS medium. The inoculum was prepared by growing a single colony of the bacteria at 37 °C for 16 h in lysogeny broth (LB) (tryptone (10.0 g/L), NaCl (5.0 g/L), yeast extract (2.0 g/L)). After the optical density (OD) of the starter culture was measured at a wavelength of 600 nm (OD_600_), a volume of the culture equivalent to a final OD_600_ of 0.04 was harvested and washed. It was then inoculated into each flask to begin cultivation. In the case where solid chicken fat was used and to prevent the growth of any contaminants that might have survived the sterilization by methanol, streptomycin was added to the medium to a final concentration of 50 μg/mL. For the cultivation involving sodium pentanoate as co-substrate to another carbon source, sodium pentanoate was added 24 h after inoculation to final concentrations ranging from 0.1 – 0.9% (w/v). The cultures were then cultivated at 37 °C with an agitation rate of 180 rpm. Cultivation time was dependent on the carbon source employed with a time of 48 h for triglycerides and 72 h for carbohydrates. Cells were harvested by centrifugation at 7140 ×g for 15 min. The supernatant was decanted and pelleted cells were resuspended in distilled water. For cells cultivated in triglycerides, cells were resuspended in equal amounts of hexane and water. Cells were then collected by centrifugation. The process was repeated twice. Lastly, the cells were resuspended in 2 mL distilled water, frozen at -20 °C and subsequently lyophilized. Weights of the dried cells were then used to calculate the biomass for each batch of cultivation.

#### 2.2.4 Qualitative and quantitative analyses of PHA

Dried cells were weighed (10–15 mg) and put into screw-cap glass tubes. Methanolysis solution (2 mL) was added followed by 2 mL of chloroform. Tubes were sealed with polytetrafluoroethylene (PTFE) tape and heated to 100 °C for 140 min in a heating block before being cooled to room temperature. Distilled water (1 mL) was added and mixed by a vortex mixer. Once the mixture has separated into two layers, the lower chloroform layer was extracted with a Pasteur pipette and transferred into glass vials containing sodium sulfate (Na_2_SO_4_) to absorb excess water. Subsequently, 0.5 mL of the chloroform mixture was then transferred into a glass vial and 0.5 mL caprylic acid methyl ester was added. The samples were then analyzed by gas chromatography using SPB™-1 capillary column (30 m × 0.25 m; Supelco) via a PerkinElmer Clarus 600 gas chromatograph with a flame ionization detector at injection temperature of 260 °C and column oven temperature of 250 °C.

#### 2.2.5 Polymer extraction and purification

Dried cells were weighed based on the amount of PHA produced to give a final amount of 50 mg of polymer. Chloroform (100 mL) was added and the mixture was stirred for 48 h. Following this, the mixture was filtered through a Whatman® Grade No. 1 filter paper and the resulting filtrate was filtered again through a 0.2 μm pore size PTFE membrane (Pall, USA). The filtrate was concentrated in a rotary evaporator under vacuum at 50 °C until a syrupy consistency was observed. The PHA polymer was precipitated by adding methanol to the concentrated solution and allowed to dry for 24 h. The precipitate was redissolved in chloroform and reprecipitated in 10 volumes of methanol. Lastly, it was allowed to dry for 24 h.

#### 2.2.6 Determination of molar mass distribution and dispersity

Molar masses of the polymers were determined by gel permeation chromatography (GPC) and their dispersion calculated thereafter. For this purpose, 5 mg of PHA polymer was dissolved in 5 mL of chloroform to obtain a solution with a concentration of 1 mg/mL. The solution was then filtered through a 0.2 μm pore size PTFE membrane into a glass vial. Samples were analyzed using an Agilent 1200 Gel Permeation Chromatography system equipped with refractive index detector and SHODEX K-802 and K-806M columns at 40 °C. Chloroform was used as eluent at a flow rate of 0.8 mL/min. Polystyrene standards with a low molar mass dispersity were used to generate the calibration curve.

#### 2.2.7 Statistical analysis

Data were analyzed by one-way analysis of variance (ANOVA) using SigmaPlot 12.0 (Systat Software). The statistical difference between groups was determined by Tukey's Honestly Significant Difference (HSD) test, with P < 0.05 indicating significance.

## 3 Results and discussion

### 3.1 Biosynthesis of PHA from simple carbohydrates

One of the carbon-containing classes of substrates used as feedstock for the biosynthesis of PHA is simple carbohydrates. In this group, where the substrates are typically called sugars, glucose is perhaps the most widely used carbon source in bacterial fermentation of PHA [27]. Here, it was used in preliminary studies involving simple carbohydrates to determine the cultivation time as well the amount required for a high production of PHA.

#### 3.1.1 Optimal cultivation time in glucose-supplemented MS medium

**Figure 1:**
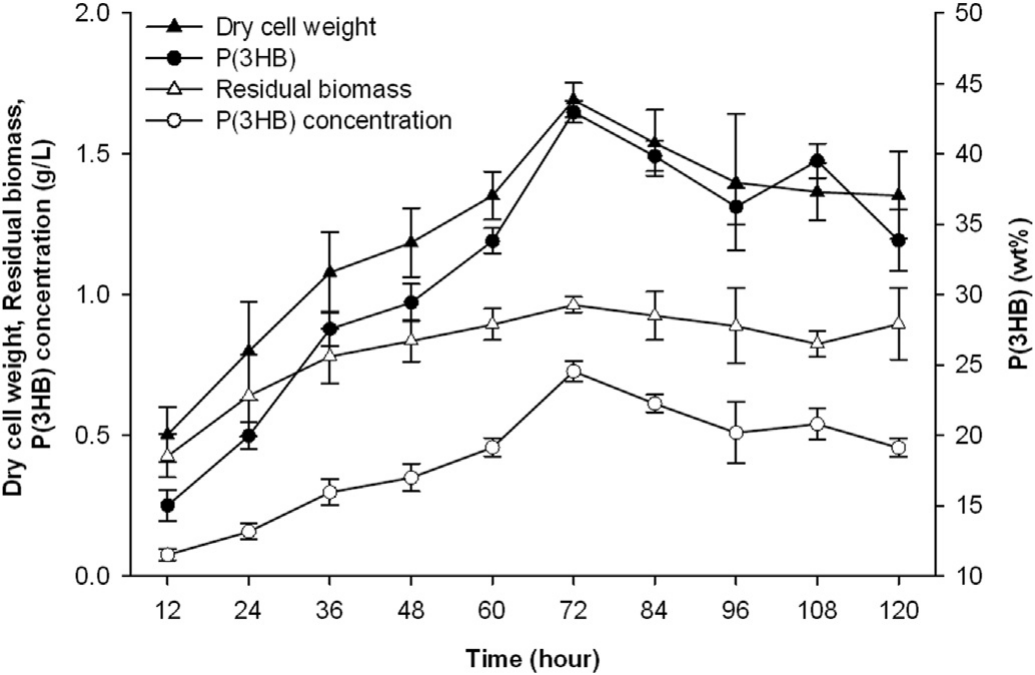
P(3HB) production, dry cell weight and residual biomass of *B. cepacia* JC-1 when cultivated in MS medium containing 2% (w/v) glucose at 37 °C. Data are presented as mean and standard deviation (mean ± SD) and are significantly different as determined via Tukey's HSD post hoc analysis, with P < 0.05 indicating significance.

Cultivation on glucose [2% (w/v)] was carried out to establish the duration wherein a high amount of PHA could be obtained. Figure 1 shows the result of cultivation over a period of 120 h. Although the differences in dry cell weight obtained from the 60th hr interval onwards were largely insignificant, a significantly higher P(3HB) content (43 wt%) and concentration (0.73 g/L) were obtained at the 72 nd h. Hence, the duration of 72 h was chosen for all carbohydrate-supplemented cultivations for the production of PHA.

#### 3.1.2 Effects of carbon to nitrogen ratio (C:N) on P(3HB) biosynthesis

**Figure 2:**
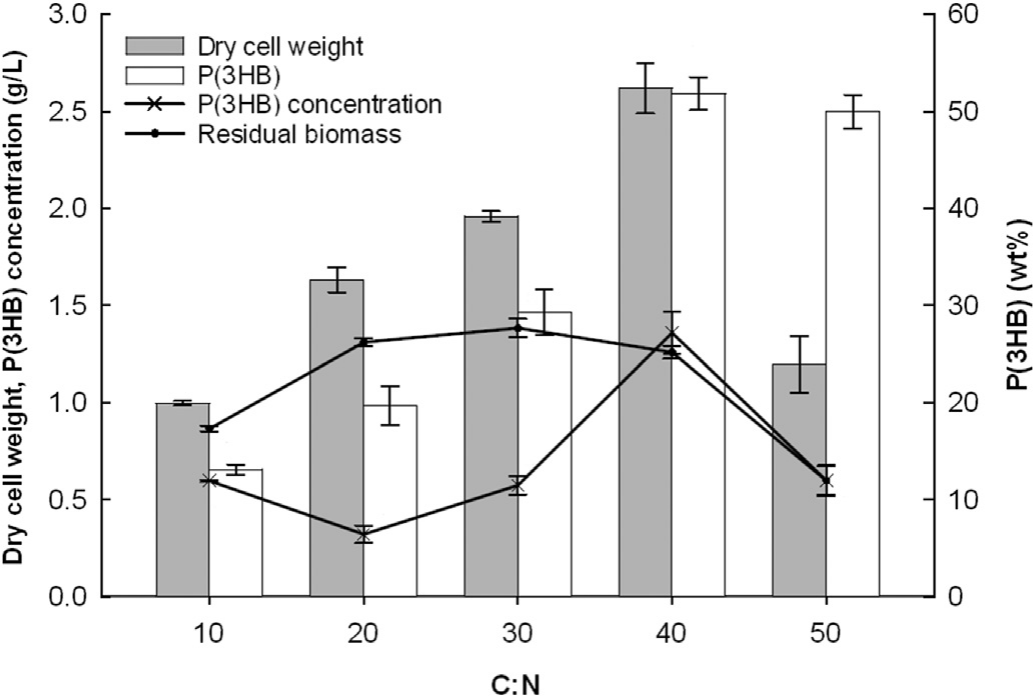
P(3HB) production, dry cell weight and residual biomass of *B. cepacia* JC-1 when cultivated in MS medium containing glucose with varying carbon to nitrogen ratios at 37 °C. Data are presented as mean ± SD and are significantly different as determined via Tukey's HSD post hoc analysis, with P < 0.05 indicating significance.

How the changes in molar ratios of elemental carbon to nitrogen affect dry cell weight, PHA production, as well as molecular weights are widely demonstrated and corroborated [28, 29, 30]. Often, many concern themselves with a maximum production of PHA and the ratios are adjusted to reflect that. Here, a range of different C:N values were employed beginning at a C:N of 10–50, with an increment of 10. From a C:N of up to 40, there was an upward trend in both dry cell weight and amount of P(3HB) produced (Figure 2). Both the dry cell weight (2.65 g/L) and P(3HB) content (52 wt%) were the highest at the C:N of 40. Consequently, the P(3HB) concentration (1.36 g/L) is significantly higher than the other batches. The dry cell weight and P(3HB) content obtained at C:N 40 is higher than those reported for *Pseudomonas pseudoflava* [31] and *Pseudomonas sp 61-3* [28] for the corresponding C:N ratio and carbon source.

On the other hand, the batch from C:N 50 gave a lower dry cell weight relative to the other batches whilst the polymer content remained approximately the same compared with the one at C:N 40. This dip in dry cell weight could be attributed to the higher concentration of glucose which in turn increases the osmotic pressure in the cultivation milieu. As the cells adapt to the changes and recover from osmotic shock, a period of lag will occur before growth and at times would continue after that [32, 33]. Hence, the amount of dry cell weight obtained at the end of cultivation will be lower. Comparing the batch cultivated at C:N 50 with the one at C:N 40, P(3HB) production did not appear to be affected and P(3HB) contents remained approximately the same. This reflects how the ability to synthesize PHA confers better tolerance toward growth-antagonizing factors in PHA-producing bacteria over their non-producing counterparts [34, 35]. However, this positive effect is said to be brought about by the normal functioning of the PHA cycle and not exclusively to the presence of the polymer [36]. Although the amount of P(3HB) at C:N 50 is comparable to that at 40, a low biomass means that the polymer concentration is effectively lower. Hence, the value of C:N 40 was chosen to be used in subsequent cultivations on other simple carbohydrates.

#### 3.1.3 P(3HB) biosynthesis from other simple carbohydrates

The cultivation on simple carbohydrates other than glucose, with the exception of lactose, supported growth and led to the accumulation of P(3HB) in the cells. As shown in Table 2, the polymer concentration obtained from each batch proceeded in the order of glucose > xylose > fructose > sucrose > galactose. The residual biomass of 2.44 g/L when cultivated on xylose is significantly higher, but with decreased polymer content. The polymer content of 51.87 wt% when cultivated on glucose was the highest compared to the other carbohydrates. Apart from glucose and galactose, there were no significant differences in the amount of P(3HB) produced. Overall, there was not a single sugar that contributed to the highest biomass and polymer content. However, if assessed from the amount of polymer produced, glucose gave a higher P(3HB) concentration (1.36 g/L) over xylose (1.03 g/L).

**Table 2:** Biosynthesis of P(3HB) by *B. cepacia* JC-1 from simple carbohydrates at C/N of 40 in MS medium, at 37 °C. Data are presented as mean ± SD and are significantly different as determined via Tukey's HSD post hoc analysis, with P < 0.05 indicating significance.

Carbohydrate	Dry cell weight (g/L)	P(3HB) content (wt%)	Residual biomass (g/L)	P(3HB) concentration (g/L)
Fructose	3.07 ± 0.08	27.37 ± 2.01	2.23 ± 0.12	0.84 ± 0.04
Galactose	2.32 ± 0.09	16.66 ± 0.78	1.93 ± 0.10	0.39 ± 0.01
Glucose	2.62 ± 0.13	51.87 ± 1.70	1.26 ± 0.03	1.36 ± 0.11
Sucrose	2.71 ± 0.07	23.17 ± 0.45	2.08 ± 0.07	0.63 ± 0.01
Xylose	3.47 ± 0.10	29.72 ± 3.28	2.44 ± 0.18	1.03 ± 0.09
Lactose	0.16 ± 0.07	Not detected	0.16 ± 0.07	-

The metabolism of these simple carbohydrates by *B. cepacia* occurs via multiple pathways: the glycolytic pathway, the pentose phosphate pathway, and the Entner-Doudoroff pathway, as determined from the Kyoto Encyclopedia of Genes and Genomes (KEGG) database [37, 38]. Although it is shown how the product of one pathway becomes the intermediate of another to ultimately yield pyruvate, the rate at which the metabolites are generated is not reflected in these reference pathways. It is thus possible that the amount of available pyruvate to be oxidized to acetyl-CoA – and subsequent channeling of the resulting flux towards PHA biosynthesis – depends on enzyme activities of the supplying pathways. This can be illustrated by the metabolism of xylose and that of galactose.

The metabolism of xylose [37] first involves the conversion of xylose to xylulose, followed by phosphorylation of xylulose to form xylulose-5-phosphate. From here onwards, xylulose-5-phosphate could directly participate in the non-oxidative phase of the pentose phosphate pathway or converted further to ribulose-5-phosphate, which is also a reactant in the same pathway. Continuation of the pathway will then yield products that will be channeled to the glycolytic and Entner-Doudoroff pathways respectively to obtain pyruvate. On the other hand, the metabolism of galactose via the Leloir pathway [39] will yield glucose-1-phosphate that will enter the Entner-Doudoroff pathway.

Although fewer enzymatic reactions occur during the metabolism of galactose [40], it appears that galactose was used as energy source and PHA biosynthesis to a lesser degree than xylose. Clearly, the robustness of each pathway differs and this leads to a different amount of product/intermediate generated for a given period.

No PHA was detected for the cultivation on lactose. There is no reason to believe that the cells could grow on lactose but not accumulate PHA. The small amount (0.16 g/L) of dry cell recorded most likely stemmed from growth from the nutrients that were carried over within the washed cells before a complete depletion finally halts cell growth.

### 3.2 Biosynthesis of PHA from triglycerides

#### 3.2.1 Optimal cultivation time for palm oil-supplemented MS medium

**Figure 3:**
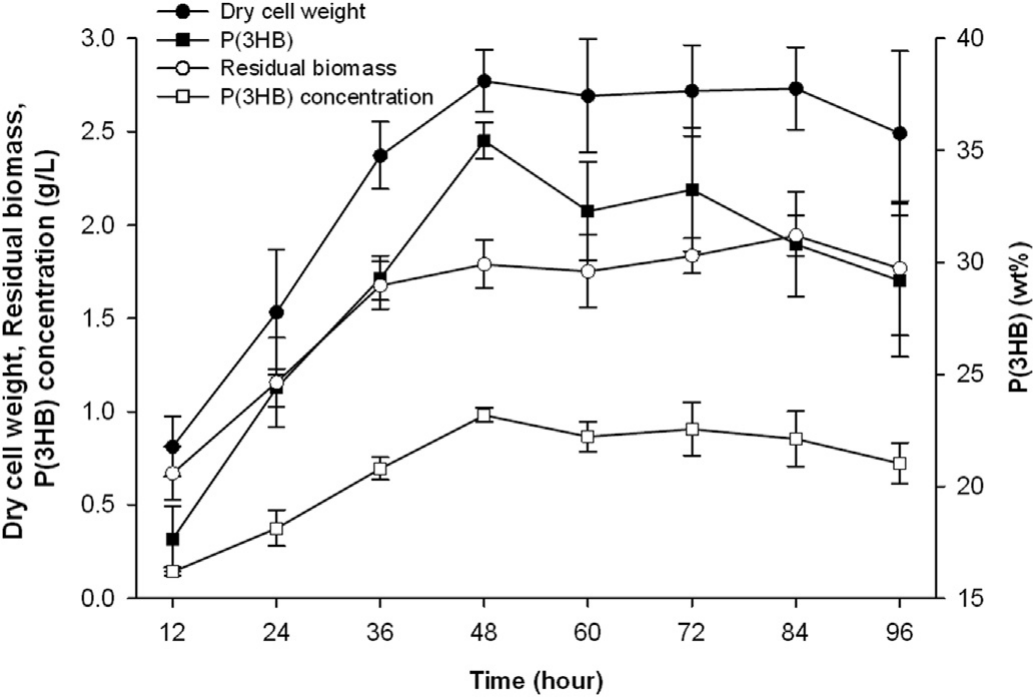
P(3HB) production, dry cell weight and residual biomass of *B. cepacia* JC-1 when cultivated in MS medium containing 2% (v/v) palm oil at 37 °C. Data are presented as mean ± SD and are significantly different as determined via Tukey's HSD post hoc analysis, with P < 0.05 indicating significance.

Apart from simple carbon sources, complex carbon sources such as plant oils were used to produce PHA and some other natural PHA producers are able to produce medium-chain-length (MCL) PHA when long chain fatty acids are used. In the case of *B. cepacia* JC-1, the cultivation time of the bacterium on triglyceride substrates was firstly determined using 2% (v/v) palm oil. Whilst there was no difference between the dry cell weight at the 24th and 48th hr, the P(3HB) content of the batch at the 48th hr was significantly higher (Figure 3). Therefore, a duration of 48 h was used for subsequent cultivations on triglycerides.

#### 3.2.2 P(3HB) biosynthesis from plant oils

In addition to palm oil, coconut oil and soybean oil were also used as the sole carbon sources for polymer biosynthesis and like the cultivation on palm oil, only P(3HB) homopolymer was detected. As shown in Table 3, utilization of plant oils resulted in the production of P(3HB) in amounts comparable to those obtained from most carbohydrates, with P(3HB) contents ranging from 25 wt% to 36 wt% from soybean oil and palm oil, respectively. Polymer concentrations are significantly higher from palm oil than those of coconut and soybean oil.

**Table 3:** Biosynthesis of P(3HB) by *B. cepacia* JC-1 from plant oil in MS medium at 37 °C. Data are presented as mean ± SD and are significantly different as determined via Tukey's HSD post hoc analysis, with P < 0.05 indicating significance.

Carbon source	Concentration [% (v/v)]	Dry cell weight (g/L)	P(3HB) content (wt%)	Residual biomass (g/L)	P(3HB) concentration (g/L)
Palm oil	0.5	3.12 ± 0.11	32.39 ± 0.90	2.11 ± 0.07	1.01 ± 0.05
	1.0	3.17 ± 0.20	32.98 ± 1.86	2.13 ± 0.19	1.04 ± 0.03
	1.5	2.83 ± 0.23	31.79 ± 2.38	1.93 ± 0.23	0.90 ± 0.01
	2.0	2.87 ± 0.10	36.07 ± 1.42	1.83 ± 0.03	1.04 ± 0.08
	2.5	2.71 ± 0.12	33.93 ± 1.56	1.79 ± 0.09	0.92 ± 0.06
	3.0	2.77 ± 0.30	33.31 ± 2.33	1.85 ± 0.19	0.92 ± 0.14
Coconut oil	0.5	1.16 ± 0.37	14.50 ± 1.09	0.99 ± 0.32	0.17 ± 0.05
	1.0	1.33 ± 0.44	18.80 ± 0.99	1.08 ± 0.35	0.25 ± 0.09
	1.5	1.19 ± 0.19	15.89 ± 2.11	1.00 ± 0.14	0.19 ± 0.05
	2.0	1.23 ± 0.68	17.37 ± 1.82	1.02 ± 0.57	0.21 ± 0.11
	2.5	1.24 ± 0.52	17.32 ± 3.25	1.01 ± 0.39	0.23 ± 0.14
	3.0	1.05 ± 0.58	15.77 ± 3.11	0.88 ± 0.49	0.17 ± 0.09
Soybean oil	0.5	2.33 ± 0.40	25.21 ± 1.37	1.74 ± 0.27	0.59 ± 0.13
	1.0	2.67 ± 0.78	29.33 ± 0.99	1.89 ± 0.55	0.78 ± 0.24
	1.5	2.39 ± 0.32	30.02 ± 1.18	1.67 ± 0.20	0.72 ± 0.12
	2.0	2.47 ± 0.25	29.59 ± 0.54	1.74 ± 0.17	0.73 ± 0.09
	2.5	2.47 ± 0.27	29.88 ± 1.48	1.73 ± 0.15	0.74 ± 0.12
	3.0	2.11 ± 0.73	31.15 ± 2.08	1.45 ± 0.49	0.66 ± 0.25

The use of triglycerides for the biosynthesis of PHA is preferred not only due to their ability to support growth, but also because of the higher amount of carbon content, weight for weight, compared to other simpler carbon sources [41]. Comparing between the amounts of PHA produced from palm oil and soybean oil, a lower value in the latter could be attributed to the extra steps involved in the breakdown of the polyunsaturated fatty acid components. Metabolism of polyunsaturated fatty acids, which is in higher percentage in soybean oil, via the beta-oxidation pathway involves a reaction catalyzed by the NADPH-dependent 2,4-dienoyl-CoA reductase [42]. Since acetoacetyl-CoA reductase (PhaB), which is one of the enzymes involved in the P(3HB) biosynthetic pathway, is also dependent on NADPH, the reduction of linoleic and linolenic acid by 2,4-dienoyl-CoA reductase might affect the production of P(3HB).

Lower amounts of residual biomass from the batch cultivation on coconut oil was most likely due to the fact that lauric acid, a predominant fatty acid in coconut oil, exerts its antimicrobial effects upon breakdown of the triacylglycerol molecules [43].

The presence of P(3HB) homopolymer after cultivation on triglycerides indicates the predilection of the strain's polymerizing enzyme, PHA synthase, for SCL-PHA regardless of the types of carbon sources used during cultivation.

Use of palm oil as feedstock for polymer production over other plant oils like soybean oil for instance, is seen as the preferred option since palm oil is superior to other oil crops in terms of annual production and efficiency [44, 45]. Whilst the use of triglycerides from edible sources for the production of PHA is pervasive, inedible oils and waste products are being given serious considerations in the past decade [46, 47], be it for bioplastics production or for other industrial processes.

#### 3.2.3 P(3HB) biosynthesis from animal fat

As the use of waste product as feedstock for the biosynthesis of PHA is much desired, laboratory-extracted chicken fat as well as the crude form were used as the carbon source for cultivation. When chicken fat was substituted for plant oil, there was a marked increase in P(3HB) content and concentration to 46.73 wt% and 1.49 g/L respectively, surpassing even that of palm oil which gave the highest amount in the group of plant triglycerides (Table 4). Apart from a similar fatty acid profile, compositionally, between the two [48], Cromwick et al. (1996) [49] posited that an additional growth-promoting factor may exist in the form of cholesterol which is present in all animal-derived lipids. The use of chicken fat is desirable since a high amount of triglyceride [50] can be extracted from the adipose tissues.

**Table 4:** Biosynthesis of P(3HB) by *B. cepacia* JC-1 from 2% chicken fat in MS medium at 37 °C. Data are presented as mean ± SD and are significantly different as determined via Tukey's HSD post hoc analysis, with P < 0.05 indicating significance.

Chicken fat	Dry cell weight (g/L)	P(3HB) content (wt%)	Residual biomass (g/L)	P(3HB) concentration (g/L)
Extracted	3.19 ± 0.02	46.73 ± 2.54	1.70 ± 0.09	1.49 ± 0.07
Extracted, with antibiotic^a^	3.03 ± 0.06	45.92 ± 2.84	1.64 ± 0.07	1.39 ± 0.11
Crude solid^a^	2.56 ± 0.27	38.76 ± 4.10	1.56 ± 0.06	1.00 ± 0.20

a Streptomycin added to a final concentration of 50 μg/mL.

The addition of streptomycin was a preventive measure against growth of contaminants during cultivation as the crude solids are at best, only surface-sterilized by methanol. Although a decrease in dry cell weight and PHA production was expected in the streptomycin-supplemented batch of extracted chicken fat – stemming from cellular processes involved to mitigate the effects of antibiotic stress [51] – there was only a slight decrease in both dry cell weight and P(3HB) content relative to the antibiotic-free batch.

**Figure 4:**
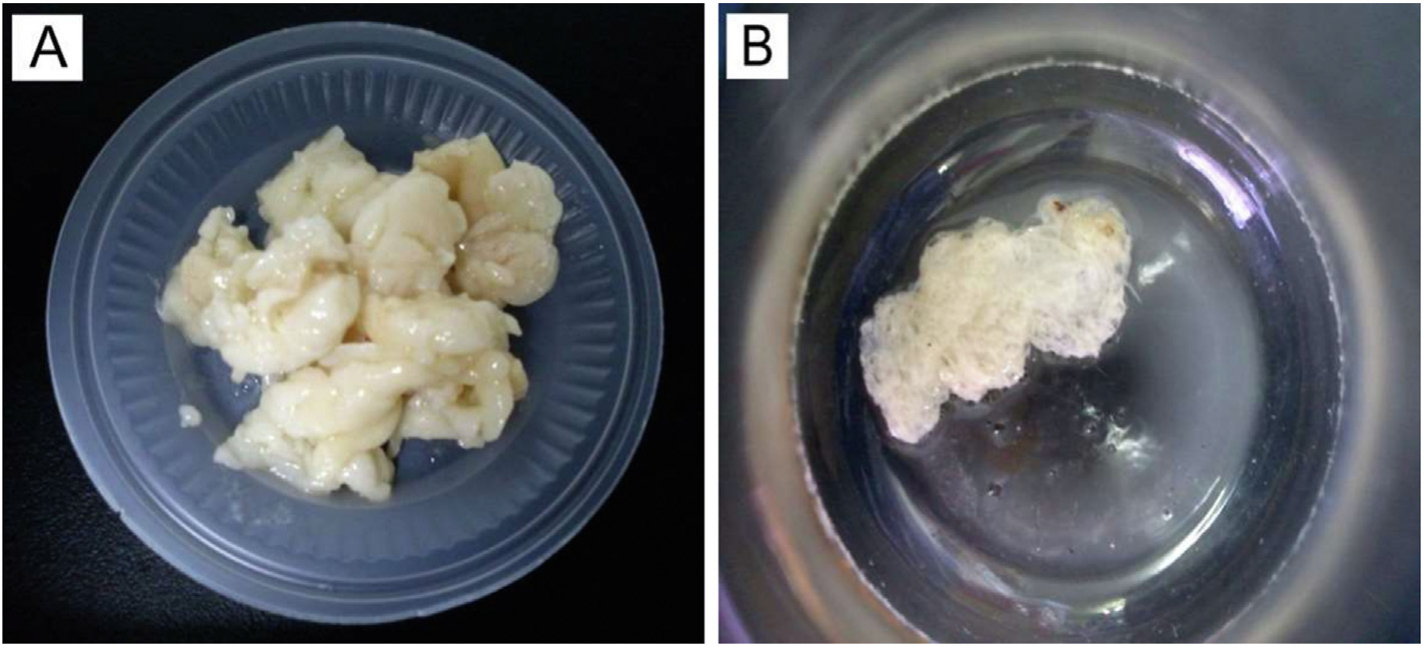
Crude chicken fat directly used in cultivation, (A) before addition into bacterial culture and (B) appearance at the end of cultivation.

This result indicates that the apparent decrease in dry cell weight (from 3.03 g/L to 2.56 g/L) and P(3HB) content (from 45.92 wt% to 38.76 wt%) for the streptomycin-supplemented batch cultivated using the crude solid fat was not caused by the antibiotic. Instead, it was due to either one or a combination of these two factors: the first being that the crude fat being used (2 g) actually contained less triglyceride than the batches cultivated on extracted fat. Another factor that could contribute to this decrease is the physical shape of the crude fat (Figure 4), increasing the time needed for the diffusion of lipase and subsequent liberation of fatty acids from within the tissues.

Polymer production was evaluated using different amounts of crude chicken fat and whilst P(3HB) contents do not differ by much (Table 5), 3 g of crude fat was found to be the better amount in relation to the others. Comparing the P(3HB) contents obtained from the use of crude chicken fat by *B. cepacia* JC-1 in this study with *P. resinovorans* on tallow [49], the latter produced a maximum polymer concentration of 0.15 g/L. The use of tallow free fatty acid to overcome low lipolytic activities of *P. putida*, *P. oleovorans*, and *P. citronellosis* resulted in polymer concentration in the range of 0.03 g/L – 0.38 g/L [49].

**Table 5:** Biosynthesis of P(3HB) by *B. cepacia* JC-1 from crude chicken fat in MS medium at 37 °C. Data are presented as mean ± SD and are significantly different as determined via Tukey's HSD post hoc analysis, with P < 0.05 indicating significance.

Amount [% (w/v)]	Dry cell weight (g/L)	P(3HB) content (wt%)	Residual biomass (g/L)	P(3HB) concentration (g/L)
1	2.94 ± 0.87	32.45 ± 1.07	1.98 ± 0.58	0.96 ± 0.29
2	2.92 ± 0.72	40.03 ± 1.11	1.75 ± 0.42	1.17 ± 0.30
3	3.47 ± 0.08	38.66 ± 0.89	2.13 ± 0.06	1.34 ± 0.04
4	3.07 ± 0.68	34.60 ± 1.43	2.01 ± 0.49	1.06 ± 0.20
5	3.27^a^ ± 0.96	34.55^a^ ± 0.63	2.14^a^ ± 0.61	1.13^a^ ± 0.35

Waste streams from the slaughtering industry are being tapped into as sources for the production of PHA. The ability of *B. cepacia* JC-1 to utilize crude fat directly due to the presence of lipase [52] eliminates the need to hydrolyze a lipid source prior to cultivation or engineer lipase-positive mutants [53] for that purpose. It is also possible to improve upon the method of cultivation using crude fat either by the addition of proteolytic enzymes such as collagenase or induction of the said enzyme during cultivation [54]. The latter could be employed for *B. cepacia* as proteases are produced by many strains that are involved in the pathogenesis of pulmonary disease in persons with cystic fibrosis [55].

#### 3.2.4 Biosynthesis of PHA from organic acids

**Figure 5:**
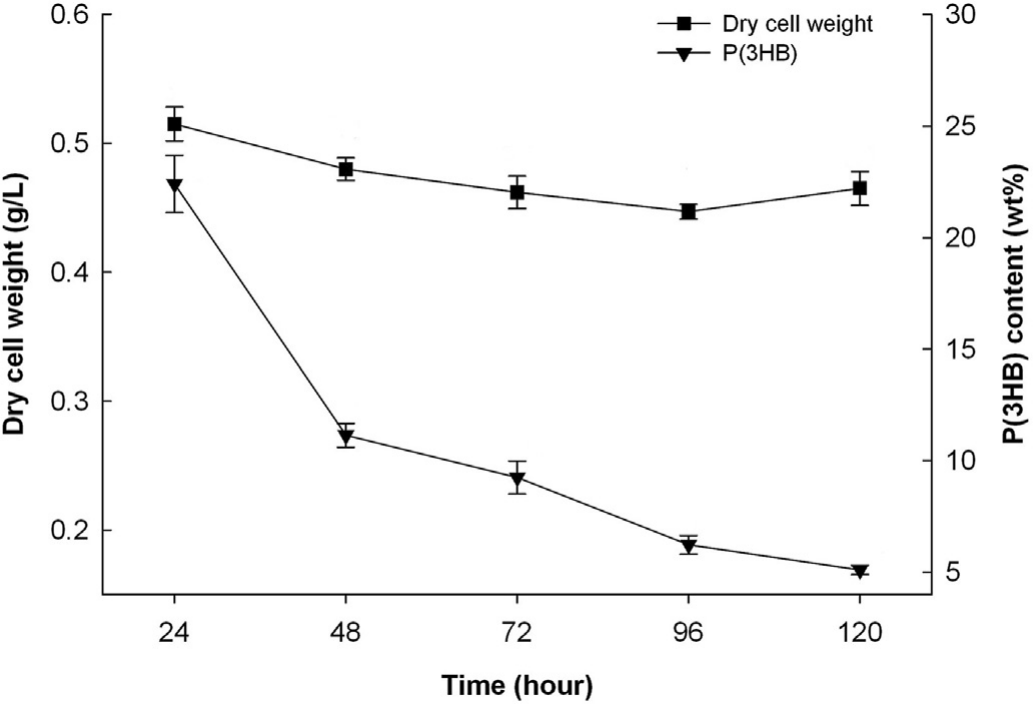
P(3HB) content and dry cell weight of *B. cepacia* JC-1 when cultivated in MS medium containing 0.1% (w/v) sodium hexanoate at 37 °C. Data are presented as mean ± SD and are significantly different as determined via Tukey's HSD post hoc analysis, with P < 0.05 indicating significance.

Biosynthesis of PHA containing constituents other than 3HB was investigated using organic acids. The use of organic acids can lead to the incorporation of structurally-related monomer units belonging to either the short-chain-length (3–5 carbon atoms) or medium-chain-length (6–14 carbon atoms) classes. Pentanoic, hexanoic, and octanoic acids, in their salt form, were used for biosynthesis of PHA. Both hexanoic and octanoic acids were used as sole carbon sources in the respective cultivation whilst pentanoic acid was added as a co-substrate with glucose. A time course growth and PHA biosynthesis for sodium hexanoate [0.1 % (w/v)] was undertaken and a cultivation time of 24 h was determined to be the most suitable period to obtain a modest amount of P(3HB) (Figure 5).

After the 24 h cultivation period, the dry cell weight showed a mostly downward trend. All in all, growth of the bacterium was largely suppressed at 0.1% (w/v) concentration of sodium hexanoate with the measured dry cell weight hovering around 0.45–0.52 g/L. As with the dry cell weight, the P(3HB) content also showed a downward trend with cultivation time with a content of 22.40 wt% at the 24th hr decreasing to 5.10 wt% at the 120th hr. With that, the cultivation time was applied for subsequent cultivation using a varied amount of organic acids, beginning first with sodium hexanoate. When an increasing concentration of sodium hexanoate (0.1%–0.6 %) was used (Table 6), the recorded dry cell weight showed an increase from 0.38 g/L to 0.75 g/L for concentrations up to 0.3% followed by a decrease beginning at 0.4%. The same trend was observed for P(3HB) content of the cells across the concentration range. Based on the dry cell weight values obtained from the various sodium hexanoate concentrations, the concentrations 0.2%–0.4% resulted in higher dry cell weight than the other concentrations, but there were no statistically significant differences between the three concentrations. However, conclusions about the concentration of sodium hexanoate that produced the highest amount of P(3HB) polymer could not be inferred from the results.

**Table 6:** Biosynthesis of P(3HB) by *B. cepacia* JC-1 from sodium hexanoate in MS medium at 37 °C. Data are presented as mean ± SD and are significantly different as determined via Tukey's HSD post hoc analysis, with P < 0.05 indicating significance.

Concentration [% (w/v)]	Dry cell weight (g/L)	P(3HB) content (wt%)	Residual biomass (g/L)	P(3HB) concentration (g/L)
0.1	0.38 ± 0.08	13.93 ± 0.79	0.33 ± 0.06	0.05 ± 0.01
0.2	0.68 ± 0.02	20.48 ± 0.79	0.54 ± 0.02	0.14 ± 0.01
0.3	0.75 ± 0.12	27.02 ± 2.34	0.55 ± 0.12	0.20 ± 0.02
0.4	0.58 ± 0.03	26.05 ± 4.25	0.43 ± 0.04	0.15 ± 0.02
0.5	0.47 ± 0.01	21.87 ± 0.61	0.37 ± 0.01	0.10 ± 0.01
0.6	0.30 ± 0.09	19.96 ± 3.15	0.24 ± 0.06	0.06 ± 0.03

Sodium octanoate was also used for cultivation and just as the case with sodium hexanoate, it resulted in poor growth (Table 7). Increasing the concentration of sodium octanoate from 0.1% to 0.2% resulted in an increase in dry cell weight. At 0.3%, growth was suppressed and at 0.4% growth was completely inhibited. Changes in the dry cell weight were less gradual than in the sodium hexanoate batches, with the concentration at 0.2% sodium octanoate yielding the highest dry cell weight of 1.09 g/L. The highest P(3HB) content (32.15 wt%) was obtained at the concentration of 0.3% sodium octanoate.

**Table 7:** Biosynthesis of P(3HB) by *B. cepacia* JC-1 from sodium octanoate in MS medium at 37 °C. Data are presented as mean ± SD and are significantly different as determined via Tukey's HSD post hoc analysis, with P < 0.05 indicating significance.

Concentration [% (w/v)]	Dry cell weight (g/L)	P(3HB) content (wt%)	Residual biomass (g/L)	P(3HB) concentration (g/L)
0.1	0.25 ± 0.02	19.66 ± 1.56	0.20 ± 0.01	0.05 ± 0.01
0.2	1.09 ± 0.06	19.94 ± 0.56	0.88 ± 0.05	0.22 ± 0.01
0.3	0.38 ± 0.04	32.15 ± 4.87	0.26 ± 0.02	0.12 ± 0.03

The toxicity of organic acids towards cell growth was clearly observed and the results supported the fact that longer-chain organic acids, in this case an eight-carbon compound, exert a more pronounced effect due to its liposolubility and membrane-specific action [56, 57]. Du *et al.* (2001) [58] reported that the presence of medium-chain-length acids such as octanoic acid and nonanoic acid inhibited the synthesis of PHA. It was speculated that these acids could negatively affect one or all three enzyme-catalyzed reactions of the PHA biosynthetic pathway. Whilst there are studies that report on the production of MCL-PHA or copolymers consisting of MCL monomers when C:6 – C:18 organic acids were supplied in cultivation, the results obtained here showed that *B. cepacia* JC-1 only produce P(3HB) in hexanoate- and octanoate-supplemented cultivations. Accumulation of PHA consisting exclusively of short-chain-length monomers by *B. cepacia* during cultivation on organic acids have been reported on a few occasions [59, 60, 61, 62, 63]. On instances where incorporation of additional monomers besides 3HB occurs in the cell, (3HV) monomers usually predominate over other types of monomers.

#### 3.2.5 Biosynthesis of PHA from sodium pentanoate as a co-substrate

The presence of 3HV monomer unit was detected in PHA produced by *B. cepacia* JC-1 when sodium pentanoate was added into the cultivation medium [64]. As only a low dry cell weight (0.46 g/L) was obtained in a rich medium [64], sodium pentanoate was employed as a co-substrate in addition to glucose for the cultivation using MS medium. Table 8 shows the effects of increasing concentrations of sodium pentanoate on dry cell weight, PHA contents, and fractions of 3HB and 3HV monomers. Despite the presence of glucose in the cultivation medium, the addition of sodium pentanoate appeared to essentially inhibit cell growth although there was a slight increase in dry cell weight until a concentration of 0.5% (w/v) before leveling off.

**Table 8:** Biosynthesis of PHA by *B. cepacia* JC-1 from sodium pentanoate as a co-substrate of glucose in MS medium at 37 °C. The amount of glucose was fixed at C/N of 40 and the amount of sodium pentanoate was varied. Data are presented as mean ± SD and are significantly different as determined via Tukey's HSD post hoc analysis, with P < 0.05 indicating significance.

Concentration [% (w/v)]	Dry cell weight (g/L)	P(3HB) content (wt%)	Residual biomass (g/L)	P(3HB) concentration (g/L)	Monomer composition (mol%) 3HB	3HV
0.1	0.40 ± 0.10	40.93 ± 11.02	0.23 ± 0.01	0.17 ± 0.09	97.94 ± 0.80	2.06 ± 0.80
0.3	0.54 ± 0.04	47.99 ± 4.79	0.28 ± 0.03	0.26 ± 0.04	98.97 ± 0.18	1.03 ± 0.18
0.5	0.73 ± 0.02	50.61 ± 3.27	0.36 ± 0.03	0.37 ± 0.02	99.37 ± 0.22	0.63 ± 0.22
0.7	0.79 ± 0.08	46.32 ± 4.44	0.42 ± 0.02	0.37 ± 0.07	99.25 ± 0.20	0.75 ± 0.20
0.9	0.73 ± 0.08	44.92 ± 5.71	0.40 ± 0.01	0.33 ± 0.08	99.33 ± 0.11	0.67 ± 0.11

As for the production of PHA, there were significant differences in the amount across the concentration range although the amount was higher than those cultivated on other organic acids such as sodium hexanoate and octanoate. This is due to the presence and utilization of glucose as the main carbon source for PHA biosynthesis. Throughout the same concentration range, the 3HB unit forms the majority of the fraction of monomer detected with 3HV present at very low levels of 1–2 mol%. Previous reports on *B. cepacia* showed that the presence of a second monomer, such as 3HV, is usually copolymerized as P(3HB-co-3HV) [61, 62, 63, 65, 66, 67].

The results obtained here closely resemble those reported by Silva et al. (2000) [66] where the 3HV fraction in some selected *B. cepacia* strains ranged from 0 to 1.5 mol%. Mutants generated from that study accumulated varying amounts of the 3HV fraction up to a value of 68 mol%. This was attributed to the efficiency of propanoate conversion to 3HV monomers. Whilst the low 3HV fractions in this study can also be attributed to the physiological efficiency of the bacterium in the conversion of pentanoate, it is possible that this phenomenon is compounded by the catabolite repression by glucose. Unfortunately, the experimental design did not allow for conjectures to be made regarding the molar fraction of 3HV in the event glucose is exhausted from the medium. Nevertheless, there is no reason to dismiss the possibility that pentanoate metabolism comes under the control of certain regulatory proteins, given that propanoate metabolism was shown to be catabolically repressed by glucose and glycerol [68].

Another evidence that lends credence to the argument that pentanoate, like propanoate, is catabolically repressed by glucose is the sensitivity of the fatty acid degradation (*fad*) regulon towards repression by catabolites such as glucose. It is only induced by long-chain fatty acids [69, 70] or during the starvation of glucose [71]. Incidentally, the products of the *fad* genes are also involved in the metabolism of pentanoate to form the 3HV monomer [72]. The 3HV detected, albeit in a small percentage, could possibly be due to transient induction of the pentanoate metabolic pathway by long-chain fatty acids originating from membrane lipids of lysed cells [71]. Following that, the substitution of glucose with a triglyceride source would be a consistent choice to possibly incur better biomass as well as 3HV fractions. Palm oil was selected as the carbon source to be used with sodium pentanoate over the other plant oils due factors outlined in Section 3.2.6.

#### 3.2.6 Biosynthesis of PHA from palm oil and sodium pentanoate

Similar to the cultivation involving glucose and sodium pentanoate, 3HV monomer was also detected when palm oil was used in place of glucose. Growth in palm oil appears to be better compared to glucose based on the residual biomass obtained (Table 9). The residual biomass was around 0.9 g/L without any significant differences across the concentration range of sodium pentanoate, implying the absence of noticeable inhibition in this batch of cultivation. A better bacterial growth on palm oil compared to glucose points to the continuation of underlying processes at the metabolic level, whereby the presence of triglycerides early in the cultivation process resulted in the induction of genes necessary for their breakdown. Coincidentally, they code for the same enzymes that are also involved in the breakdown of pentanoate [72]. Thus, this priming of the metabolic system could have enabled an efficient uptake and metabolism of pentanoate the moment it is introduced into the cultivation milieu. In contrast, the presence of glucose causes no such induction when cultivated with an alkanoate source. As shown by Page and Manchak (1995) [73], when studying how alkanoates affect copolymer synthesis in *Azotobacter vinelandii* UWD, the uptake of pentanoate is not repressed in the presence of glucose. The newly acquired pentanoate molecules would accumulate whilst expression of necessary genes takes place. Hence, persistence of the pentanoate anion in the bacterial cytoplasm will cause exertion of its toxic effect [74] foremost over any subsequent palliating processes.

**Table 9:** Biosynthesis of PHA by *B. cepacia* JC-1 from sodium pentanoate as a co-substrate of palm oil in MS medium at 37 °C. The co-substrate concentration was varied and the total amount of both carbon sources was fixed at 1%. Data are presented as mean ± SD and are significantly different as determined via Tukey's HSD post hoc analysis, with P < 0.05 indicating significance.

Co-substrate concentration [% (w/v)]	Dry cell weight (g/L)	PHA content (wt%)	Residual biomass (g/L)	PHA concentration (g/L)	Monomer composition (mol%) 3HB	3HV
0.1	2.43 ± 0.06	62.92 ± 0.12	0.90 ± 0.02	1.53 ± 0.04	84.63 ± 0.19	15.37 ± 0.19
0.3	1.63 ± 0.02	39.31 ± 0.11	0.99 ± 0.01	0.64 ± 0.00	76.73 ± 0.11	23.27 ± 0.11
0.5	1.05 ± 0.06	6.93 ± 0.06	0.98 ± 0.06	0.07 ± 0.00	63.53 ± 0.29	36.47 ± 0.29
0.7	0.99 ± 0.02	8.75 ± 0.05	0.90 ± 0.02	0.09 ± 0.00	87.41 ± 0.22	12.60 ± 0.22
0.9	1.00 ± 0.04	9.60 ± 0.05	0.90 ± 0.04	0.10 ± 0.00	91.88 ± 0.18	8.12 ± 0.18

The proportions of 3HV increased as the amount of co-substrate in the growth media increased, peaking at 0.5% (w/v) sodium pentanoate. Beyond this concentration, 3HV fractions decreased to approximately 8 mol%. This observation, together with the fact that PHA content decreases as pentanoate concentration increases, suggests that the amount of co-substrate could be the factor. When used alone in cultivation, pentanoate will be largely channeled towards metabolism for growth instead of polymer production [73]. Thus, the exhaustion of the main carbon source by *B. cepacia* JC-1 particularly in cultivations with low triglyceride concentration and high co-substrate concentration could create a condition wherein the pentanoate becomes the only carbon source available for uptake. Whilst fluxes from alkanoate metabolism towards growth have been demonstrated for other bacteria [73, 75], the same might occur for this cultivation.

#### 3.2.7 Biosynthesis of PHA from chicken fat and sodium pentanoate

Like palm oil, chicken fat has served the P(3HB) biosynthesis process comparatively well and was used for the same purpose with sodium pentanoate as a co-substrate. Using 3% (w/v) of crude chicken fat with varying sodium pentanoate concentrations resulted in the biosynthesis of PHA with 3HV monomer fractions within a relatively narrower range of about 5 mol% - 14 mol% compared to the palm oil batch (Table 10). Polymer contents decreased in a more gradual fashion with increasing pentanoate concentrations. Promotion of polymer biosynthesis by amino acids in small amounts has been established [76, 77]. Either the breakdown of connective tissues of crude chicken fat or the denaturation of lipid-protein complex in animal tissues [25, 78] or both would lead to the presence of amino acids. Therefore, the gradual decrease in polymer content could possibly be due to the presence of amino acids offsetting the inhibitory effect of pentanoate on polymer biosynthesis.

**Table 10:** Biosynthesis of PHA by *B. cepacia* JC-1 from sodium pentanoate as a co-substrate of crude chicken fat in MS medium at 37 °C. The amount of crude chicken fat was fixed at 3% of the total volume of the cultivation medium and the amount of the co-substrate was varied. Data are presented as mean ± SD and are significantly different as determined via Tukey's HSD post hoc analysis, with P < 0.05 indicating significance.

Co-substrate Concentration [% (w/v)]	Dry cell weight (g/L)	PHA content (wt%)	Residual biomass (g/L)	PHA concentration (g/L)	Monomer composition (mol%) 3HB	3HV
0.1	2.59 ± 0.06	50.54 ± 0.21	1.28 ± 0.04	1.31 ± 0.02	89.48 ± 0.14	10.52 ± 0.14
0.3	1.45 ± 0.02	33.73 ± 0.17	0.96 ± 0.02	0.49 ± 0.01	86.26 ± 0.11	13.74 ± 0.11
0.5	1.32 ± 0.04	23.38 ± 0.19	1.01 ± 0.03	0.31 ± 0.01	88.99 ± 0.05	11.01 ± 0.05
0.7	0.84 ± 0.04	10.13 ± 0.06	0.75 ± 0.04	0.09 ± 0.00	91.77 ± 0.21	8.23 ± 0.21
0.9	0.99 ± 0.05	25.20 ± 0.06	0.74 ± 0.00	0.25 ± 0.01	95.54 ± 0.03	4.46 ± 0.03

The use of chicken fat for the biosynthesis of PHA mirrors the trend towards utilizing renewable and inexpensive resources to support production. The ability of this isolate to breakdown chicken fats, hence unmediated use of crude fat, removes the need for additional costs. That together with intrinsic resistance to antimicrobials of this isolate (Table 11) would reduce additional manipulations should antibiotic selection pressure be needed in any cultivation milieu involving the direct use of crude materials.

**Table 11:** Growth of *B. cepacia* JC-1 on LB agar containing antibiotics at different concentrations.

Antibiotics	Final concentration in agar (μg/mL)^a^		
	25	50	100
Ampicillin	+	+	+
Amikacin	+	+	–
Chloramphenicol	–	–	–
Ciprofloxacin	–	–	–
Kanamycin	+	–	–
Neomycin	+	+	–
Streptomycin	+	+	+
Tetracycline	+	+	+

a ‘+’ sign denotes growth whilst ‘–’ sign denotes otherwise.

### 3.3 Molecular weights of polymers produced by *B. cepacia* JC-1

The molecular weights of the polymer produced from carbon sources used in cultivation were represented by glucose, palm oil, chicken fat, and sodium hexanoate (Table 12). The molecular weights were in the range of 152–2118 kDa (weight-average, M_w_) and 71.5–390 kDa (number-average, M_n_), with the molecular weight dispersity ranging from 2.1 – 5.4. The average weights (M_w_: 2118 kDa; M_n_ 390 kDa) and the dispersity (5.4) of the polymer produced from glucose were the highest followed by those from the triglycerides, which were comparable in their values. The M_w_ of polymers synthesized from glucose and triglycerides are similar to those synthesized by *C. necator*, ranging 500 kDa–1000 kDa [79] and are higher than those produced by some recombinant strains [80, 81]. In addition, polymers produced by *B. cepacia* JC-1 from triglycerides are higher than those by *P. putida* from coconut oil (464 kDa) and lard (652 kDa) [53], by *P. resinovorans* from tallow (194 kDa) [49], and slightly lower (1032 kDa) than *C. necator* on olive oil [82]. Whilst the polymers are not within the range of ultra-high molecular weight polymers of 3000 kDa–11000 kDa [83], higher molecular weights are desirable for the purpose of industrial-level manipulations [79].

**Table 12:** Molecular weights of P(3HB) produced by *B. cepacia* JC-1 from different carbon sources.

Carbon source	M_w_^a^ (×10^3^ Da)	M_n_^b^ (×10^3^ Da)	Ð_M_^c^
Glucose	2118	390	5.4
Palm oil	991	260	3.8
Chicken fat	1099	269	4.1
Sodium hexanoate	152	71.5	2.1

a Weight-average molecular weight.

b Number-average molecular weight.

c Molecular weight dispersity, Ð_M_ = M_w_/M_n_.

These results reflect how the different classes of substrates – in this case, simple carbohydrates and triglycerides – play a role in effecting a change in molecular weights of the polymer; in keeping with results from the work by Taidi *et al.* (1994) [84]. Previous studies have shown that the hydroxyl groups in glycerol are involved in the chain-terminating reactions resulting in PHA with glycerol end-groups [85, 86].

In addition to that, a decrease in polymer molecular weight was observed when carbon sources containing at least a hydroxyl group were used during cultivation [85, 87]. Besides the role played by chain transfer agents in the regulation of molecular weight, other governing factors include the type of PHA synthase, presence of depolymerizing enzymes, amount of PHA synthase expressed and the activity of the synthase [88, 89]. Since a single type of bacterium was used with the same cultivation parameters in this study, it appears that those factors do not account for the difference in molecular weights. In the case of PHA depolymerase which is present in natural producers, the action of the enzyme should cause a decrease in molecular weight with time but the results did not indicate such an occurrence here, even when the cultivation period for those on glucose were longer than those of triglyceride. The work by Budde *et al.* (2010) [90] on PhaB draws the connection between a fall in the synthesis of (*R*)-3-hydroxybutyryl-CoA and a lower molecular weight polymer. It is thus possible that the depression of PhaB activity occurs during breakdown of polyunsaturated fatty acids catalyzed by the NADPH-dependent 2,4-dienoyl-CoA reductase, as discussed in Section 3.2.6.

The molecular weight of the batch cultivated on hexanoate was lower compared to those from the triglycerides and glucose batches. One possible reason is the cultivation duration before cell harvesting. The duration of 24 h, when compared with 48 (triglycerides) and 72 h (glucose), inevitably results in a lower molecular weight due to the shorter amount of time available for polymerization. Yet another likely explanation to this observation revolves around the amount of substrate generated by the cells for polymer biosynthesis when cultivated on sodium hexanoate. Apparently, the other classes of carbon source were better tolerated and metabolized, which led to an increase in the pool of available substrates. As asserted by Dennis *et al.* (1998) [91], an increase in available substrate for the PHA synthase will result in polymers having higher molecular weights. Relatively low molecular weight dispersity from hexanoate could simply be due to the absence of any hydroxyl group in that compound that would allow participation in chain termination events. This leads to a rather low dispersion in the distribution of the polymer chains.

## 4 Conclusion

In this study, a local isolate identified as *B. cepacia* JC-1 was found to be capable of producing PHA from a variety of renewable carbon sources. Among simple carbons and plant oil used, cultivation on glucose gave the highest amount of P(3HB), followed by palm oil. Interestingly, *B. cepacia* JC-1 showed versatility in utilizing crude chicken fat for PHA biosynthesis, resulting in PHA content surpassing even that of palm oil. Addition of sodium pentanoate as co-substrate resulted in the accumulation of P(3HB-co-3HV) polymers. It may be possible to further improve cultivation conditions and the strain for future scale up production of PHA using crude chicken fat as the main feedstock. Whilst the ability to produce MCL PHA by the strain is somewhat limited, improvement of the strain or heterologous expression of PHA synthase of *B. cepacia* JC-1 in bacterial hosts known to produce MCL-PHA would be merit of investigation. All in all, the results from this study showed how the unmediated use of crude materials can have beneficial cost-saving outcomes. It is hoped that many will aspire to a shift in focus in this field from costly manipulations and toward a more natural means of producing PHA.

## Declarations

### Author contribution statement

Julian Hock-Chye Chin: Conceived and designed the experiments; Performed the experiments; Analyzed and interpreted the data; Wrote the paper.

Mohd Razip Samian: Contributed reagents, materials, analysis tools or data.

Yahaya M. Normi: Conceived and designed the experiments; Analyzed and interpreted the data; Contributed reagents, materials, analysis tools or data; Wrote the paper.

### Funding statement

Julian Hock-Chye Chin and Yahaya M. Normi was supported by 10.13039/501100007208Malaysia Toray Science Foundation Grant 699 (304/PBIOLOGI/650464/M126).

### Data availability statement

Data included in article/supplementary material/referenced in article.

### Declaration of interests statement

The authors declare no conflict of interest.

### Additional information

No additional information is available for this paper.
